# Anatomy of the Urethra

**Published:** 1851-07

**Authors:** 


*Anatomy of the Urethra.*—Mr. Hancock made the following communication:—Assisted by Mr. Hogg, he had examined, microscopically, the structures surrounding the urethra. He found that the urethra itself was not muscular, but closely invested by organic muscular fibres continuous with the muscular coat of the bladder, which consisted of two layers, an internal and an external. The external passes forwards, and extends over the outside of the prostate gland; the internal, on the contrary, accompanies the mucous lining through the prostate, forming an involuntary muscular covering to the urethra in its passage through the gland. The membranous portion of the urethra is next closely invested by these muscular fibres, which cannot be mistaken for any portion of Wilson's, Guthrie's, or Santorini's muscles, the latter being voluntary, and presenting the usual striated appearance under the microscope, whilst the former are involuntary and nucleated. Continued forwards, they reach the bulb, where they divide, the one part being continued onwards to the orifice of the urethra, lying between it and the corpus spongiosum, whilst the other passes on the outer surface of the corpus spongiosum, separating it from its fibrous investment, to which the fibres adhere pretty closely. These latter are also continued forward to the orifice of the urethra, and, in their course, invest the spongy portion of the bulb, uretha, and glans penis, entering very largely into the formation of that peculiar structure found at the orifice of the urethra, and which appears to consist almost entirely of involuntary muscle and elastic cellular tissue, forming an additional sphincter muscle to those usually described as met with in various parts of the body. It will thus be seen that the corpus spongiosum urethra lies between two layers of involuntary muscle, the one separating it from the urethra, the other from its fibrous covering; an arrangement, doubtless, exerting considerable influence over the expulsion of the blood from the corpus spongiosum when erection of the organ is no longer required, as well as over the emission of urine and the seminal fluid. After the meeting,

Mr. Hancock exhibited, under the microscope, specimens of the muscular tissue just described.—*Ibid.*

